# Supplementary figures and images for: Systems Biology Approach to Model the Life Cycle of Trypanosoma cruzi
Source: PLoS One. 2016 Jan 11;11(1):e0146947. doi: 10.1371/journal.pone.0146947 (PMC4709001; doi:10.1371/journal.pone.0146947)

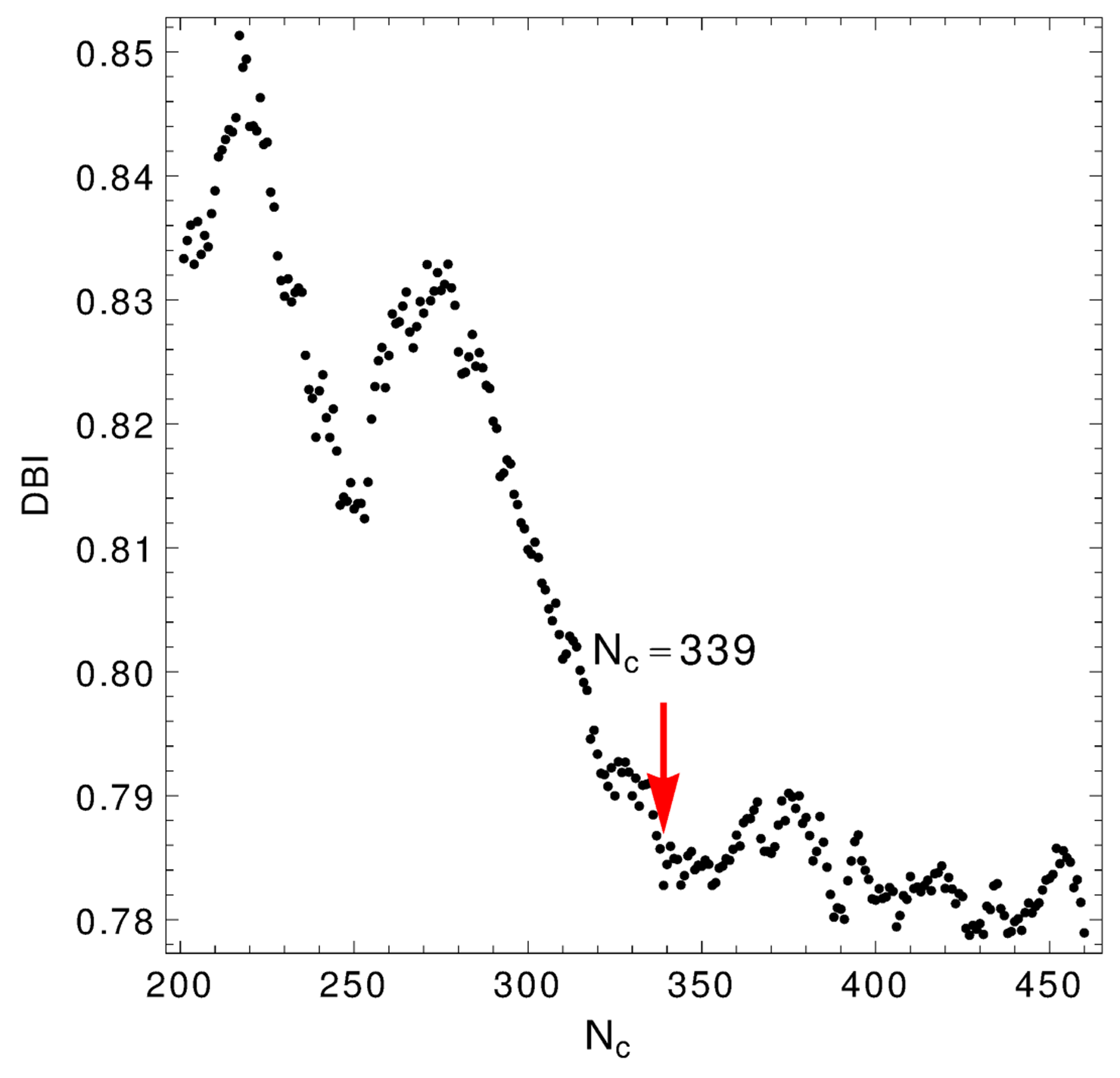

Supplement: S1 Fig — Davies-Bouldin index (DBI) as a function of the number of clusters, Nc, used in the clustering procedure. The arrow in Nc = 339 indicates the optimal number of clusters used in subsequent procedures. (TIF) [file pone.0146947.s001.tif]

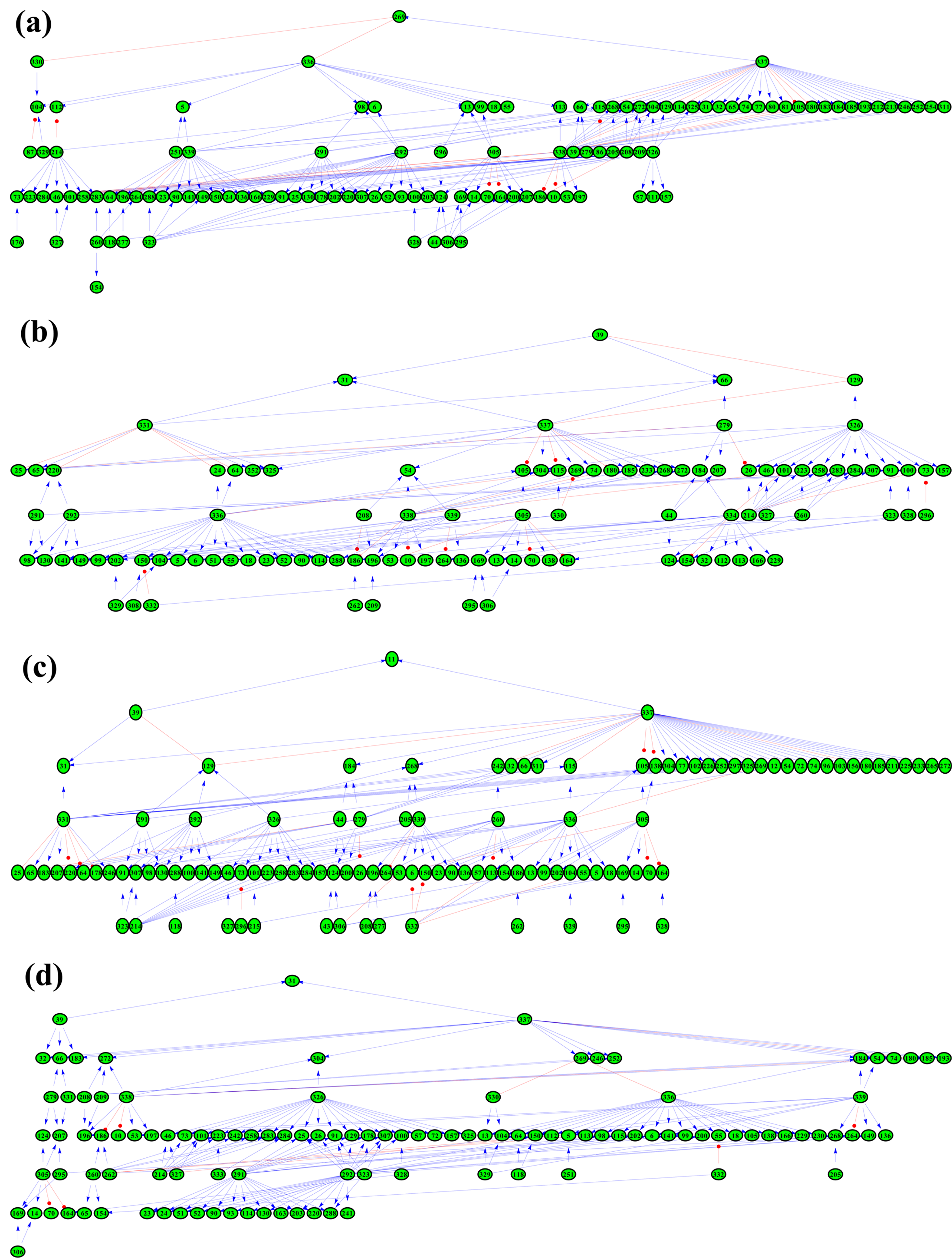

Supplement: S2 Fig — The plots represent the networks derived from amastigote (a), epimastigote (b), metacyclic tryp. (c), and trypomastigote (d) stages. (TIF) [file pone.0146947.s002.tif]

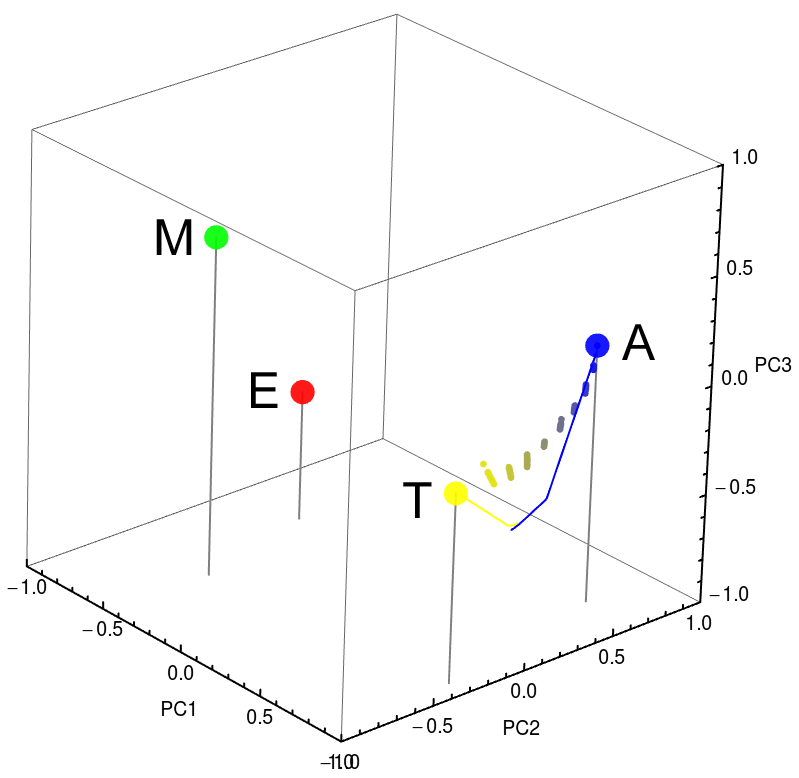

Supplement: S3 Fig — Yellow trajectory shows the dynamics of the system, in the space spanned by the three principal components, when steady state corresponding to the trypomastigote stage is perturbed by mean of over-expressing and knocking-down genes belonging to cluster 326 and 337, respectively. Blue trajectory shows the dynamics of the system when clusters 259, 260, and 332, predicted to be down-regulated by the external cue leading to the trypomastigote stage, are overexpressed. For comparison, trajectories corresponding to the unperturbed transition from amastigote to trypomastigote stages are also plotted (colored circles). (TIF) [file pone.0146947.s003.tif]
